# Characterization of serotonin-5-HTR1E signaling pathways and its role in cell survival

**DOI:** 10.21203/rs.3.rs-2518076/v1

**Published:** 2023-01-27

**Authors:** Vinay Kumar Sharma, Kiersten Campbell, Xuyu Yang, Ryan Dale, Y. Peng Loh

**Affiliations:** 1 Section on Cellular Neurobiology, *Eunice Kennedy Shriver* National Institute of Child Health and Human Development, National Institutes of Health, Bethesda, MD 20892, USA.; 2 Bioinformatics and Scientific Programming Core, *Eunice Kennedy Shriver* National Institute of Child Health and Human Development, National Institutes of Health, Bethesda, MD 20892, USA.

**Keywords:** GPCR, G proteins, cell signaling, cell survival

## Abstract

5-Hydroxy tryptamine receptor 1E (5-HTR1E) is reported to activate cAMP and ERK pathways *via* its ligands and binding partners, but the detailed mechanism underlying the serotonin induced 5-HTR1E signaling is still not known. In the present study, we determined the cellular regulators of ERK and cAMP signaling pathways in response to serotonin induced 5-HTR1E activation in 5-HTR1E overexpressing HEK293 cells. We found that Pertussis Toxin (PTX) treatment completely reversed the effect of serotonin-5-HTR1E mediated signaling on cAMP and ERK pathways, confirming the involvement of a Gαi-linked cascade. We also observed that Gβγ and Gq were not associated with 5-HTR1E activation, while blocking PKA inhibited ERK signaling only, and had no effect on cAMP. Additionally, serotonin-stimulated ERK1/2 phosphorylation was similar in 5-HTR1E overexpressing, β-arrestin-deficient HEK293 cells and is solely dependent on G protein signaling. siRNA mediated gene knockout studies in SH-SY5Y cells revealed that the inhibition of 5-HTR1E reduced the expression of *cMyc*, *Cyclin D1*, *Cyclin E* and *BCL2* genes which are related to cell cycle regulation and survival. MTT assays showed that 5-HTR1E knockdown in SHSY-5Y and U118 cells inhibited cell survival significantly. In addition to the signaling mechanism, we also performed RNA-seq analysis in 5-HTR1E overexpressing HEK293 cells and found that 5-HTR1E can regulate the expression of Receptor activity modifying protein 1 (*RAMP1*), Nuclear receptor 1 (*NR4A1*) and other Cyclin genes. These findings indicate that serotonin interaction with 5-HTR1E receptor simultaneously activates cAMP and ERK pathway in HEK293 cells and its expression is important for cell survival.

## Graphical Abstract

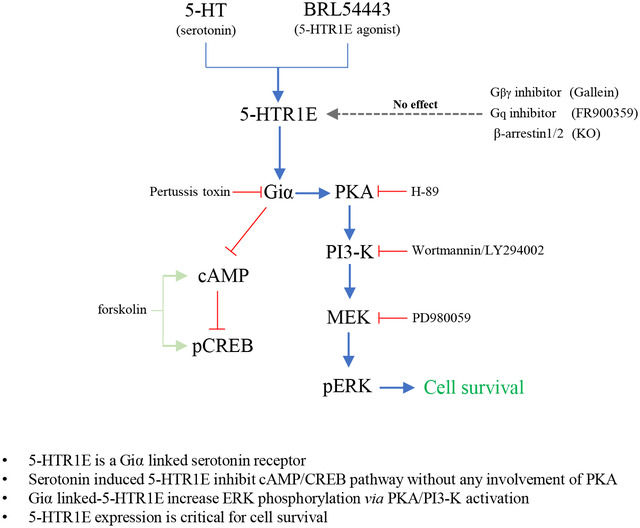

## Introduction

Serotonin, a monoamine neurotransmitter has thirteen mammalian receptors identified so far. These serotonin receptors are categorized into seven major classes, 5-HT1 to 5-HT7. All serotonin receptors belong to G protein-coupled receptor (GPCR) family except 5-HT3 which is ligand-gated ion channels (1). The largest serotonin receptor family 5-HT1 consists of five members named as 5-HTR1A, 5-HTR1B, 5-HTR1D, 5-HTR1E and 5-HTR1F (2). Several studies done on these 5-HT1 family receptors have shown that they can contribute to various physiological processes like neuronal regulation (3), neuroprotection (4) and ailments like pain, CNS disorders (5, 6), migraine (7) and cancer (8, 9). Despite the enormous work done on 5-HT1 receptors, 5-HTR1E is the least explored serotonin receptor in this family.

Like the other 5-HT1 members, 5-hydroxytryptamine receptor 1-E (5-HTR1E) is also a Gαi-coupled receptor which inhibit cellular cAMP level (1). The 5-HTR1E gene is located on human chromosome 6q14-q15, and does not contain any introns (10). 5-HTR1E is absent in rats and mice but is expressed in guinea pigs, monkeys, and humans (11–14). In terms of function, earlier reports which identified 5-HTR1E as a fifth member of 5-HT1 family were only limited to 5-HTR1E mediated cAMP inhibition. Distribution studies showed the high expression of 5-HTR1E in hippocampus, frontal cortex, and olfactory bulb of guinea pig brain and presumed that it could have a role in memory and learning (14, 15). Since its discovery in 1989, until recently, specific functions of 5-HTR1E largely remain unknown. We recently reported the expression of 5-HTR1E in human hippocampus where it co-localizes with NFα1/CPE. 5-HTR1E can also protect the neurons against oxidative stress *via* extracellular interaction with NFα1/CPE which is a binding partner of 5-HTR1E (4). In another report, studies in human ovarian cancer SKOV3 cells showed that 5-HTR1E might be able to prevent the stress-promoted progression of ovarian cancer (9).

Even after these reports, specific serotonin/5-HTR1E signaling mechanism and downstream functions remain unexplored. In this study, we investigated in detail the signaling mechanism of 5-HTR1E with its natural ligand serotonin (5-HT) in 5-HTR1E overexpressing human embryonic kidney HEK293 cells using various cell and molecular biology assays. Additionally, we also report the effect of 5-HTR1E KO on cell cycle related genes and survival of neuroblastoma SH-SY5Y and glioblastoma U118 cells using as models. To investigate the effect of 5-HTR1E overexpression on gene expression profile, we performed RNA sequencing experiments in 5-HTR1E-HEK293 cells in the presence and absence of serotonin and analyzed the effect of 5HTR1E overexpression and serotonin interaction on many genes related to different pathways.

## Materials and method

### Cell culture

HEK293 cells stably expressing the human 5HTR1E or empty vector were obtained from Dr. Bryan Roth’s laboratory at UNC, North Carolina, USA. These cells were maintained in DMEM supplemented with 10% serum (FBS) and antibiotics. Human neuroblastoma cell line (SH-SY5Y) was purchased from the ATCC (Manassas, VA, USA) and cultured in Eagle’s minimal essential medium/F12 containing 10% FBS.

### 5-HTR1E and β-Arrestin Knockdown

SH-SY5Y cells were infected with 40 MOI Ad-5-HTR1E shRNA or control Ad-LacZ (Vector Biolabs) for 72 h in serum free media. U118 cells were transfected with human 50 nM siControl or siHTR1E (sc-42227, Santacruz biotech) using Lipofectamine^™^ RNAiMAX reagent (Cat. 13778150, Thermo scientific) for 48 h. For β-arrestin knockout, 5-HTR1E stable HEK293 cells were transfected with 50 nM human β-Arrestin1 (sc-29741) and β-Arrestin2 siRNA (sc-29208, Santacruz biotech) for 48 h.

### Quantitative real-time PCR (qPCR)

Total RNA was isolated from a 6 well plate using the RNAeasy mini kit (Qiagen). 1μg RNA was reverse transcribed using SensiFAST cDNA Synthesis Kit (cat. no. BIO-65054). Gene specific primers were designed using Primer 3 software and sequence details of these primers are provided in supplementary table 1. PCR reactions were setup in 96 well plates using SYBER green in a Quant Studio (TM) 6 Flex PCR machine (Applied Biosystems, Foster City, CA, USA). qPCR was done with cycling conditions as: 95°C for 10 min, followed by 40 amplification cycles of 95°C for 10 min, 95°C for 15 sec, 60°C for 1 min and 72 °C for 30 s. Threshold values (Ct) were determined by the Quant Studio Real-Time PCR Software and relative mRNA expression was calculated by the comparative ΔΔCt method. Data were normalized to housekeeping gene β-actin, 18S or GAPDH as specified in figure legends.

### Western blot

Cells were lysed in Pierce^™^ IP Lysis Buffer (cat. No. 87787, Thermo scientific, USA) and proteins were quantified using Bradford reagent (BioRad, USA). Total 30 μg proteins were loaded on 4–12% SDS-PAGE gel and transferred onto a nitrocellulose membrane. Blotting membranes were labelled with the protein specific antibodies (details in supple. table 2) and scanned by Odyssey infrared imaging system (Lincoln, NE) and protein bands were visualized by LI-COR Inc software. For the quantification of protein bands Image J software (NIH, Bethesda) was used.

### ERK phosphorylation

5-HTR1E stable and HEK293 control cells were seeded in 12 well plate (60–70% confluency) in DMEM media supplemented with 10% FBS and incubated overnight at 37°C in a CO_2_ incubator. Next day media was changed to serum free medium (SFM) and after 3h. cells were treated with 0 to 1 μM serotonin (5-HT, cat. no. H9523, Sigma-Aldrich) or 5-HTR1E agonist, BRL54443 (cat. no. 1129, Tocris Bioscience) for 4–7 min. For inhibitor experiments, before treatment with 1 μM 5-HT or BRL54443, control and 5-HTR1E expressing HEK293 cells were treated with 200 ng pertussis toxin (Gαi inhibitor, PTX, cat no. 516560, Sigma-Aldrich) for 24 h., 1μm FR900359 (Gq inhibitor, Institute of Pharmaceutical Biology, University of Bonn), 10 μM H-89 (PKA inhibitor, cat. no. 2910, Tocris Bioscience), PI3K inhibitors, 10 μM LY294002 (cat. no. 440202, Sigma-Aldrich) or 0.5 μM Wortmannin (cat. no. W1628, Sigma-Aldrich) for 30 min, 10 μM Gallein (G_βϒ_ inhibitor, cat. no. 3090, Tocris Bioscience) and 5 μM PD980059 (MEK inhibitor, cat. no. 1213, Tocris Bioscience) for 45 min. Change in pERK was assessed by probing the western blot membranes with pERK1/2 (Thr202/Tyr204) rabbit antibody and tERK1/2 mouse monoclonal antibody simultaneously. Fluorescence labelled anti-rabbit (800 nm) and anti-mouse (680 nm) secondary antibodies (1:10000) were used to visualize the protein bands. pERK was normalized with tERK, and fold change were calculated from three independent experiments after densitometric analysis using image J software, NIH.

### cAMP assay

The cAMP assay was performed in 5-HTR1E expressing HEK293 cells. 10,000 cells/well were seeded in a lysine coated 96 well plate in 10% FBS DMEM media. On the next day, cells were incubated with 200 ng PTX (Gi inhibitor) in serum free media for 24 h. or 10 μM H-89 (PKA inhibitor) for 30 min. Before treatment with 1μM 5-HT or BRL54443, these cells were induced with 10 μM forskolin (cAMP activator) in the presence of phosphodiesterase inhibitor (Cat. no. 524718, set I-Calbiochem, Sigma Aldrich, USA) for 15 min. The cAMP assay was performed using cAMP-Glo^™^ Assay kit (cat. no. V1501, Promega, USA) according to the manufacturer’s protocol and luminescence was recorded on plate reader. All experiments were performed in triplicate and repeated at least three times.

### CREB phosphorylation

5-HTR1E stable cells were seeded in 12 well plate (1×10^5^/well) in complete DMEM. On the next day, in SFM media cells were treated with 1μM 5-HT in the presence and absence of 10 μM forskolin for 20 min. Changes in pCREB levels were assessed by western blot using pCREB rabbit antibody against Ser133 and tCREB mouse antibody. Control HEK293 cells were also analyzed for 5-HT mediated CREB phosphorylation.

### MTT assay

SHSY-5Y cells were seeded in 35 mm dishes and transduced with 40 MOI AdControl or AdHTR1-shRNA. After 48 h. these cells were seeded in a 96-well plate at a density of 2000 cells/well and treated with or without 1μM 5-HT. For MTT assay in glioma cells, U118 cells transfected with 50 nM 5-HTR1E siRNA (Santa Cruz Biotechnology Inc) or control siRNA for 48 h. and an MTT (3-(4,5-dimethylthiazol-2-yl)-2,5-diphenyltetrazolium bromide) assay was performed on days 1, 3, 5, and 7. Briefly, 25 μl of MTT reagent (5 mg/ml, Sigma-Aldrich) was added per well and incubated at 37°C in a CO_2_ incubator for 4h. Supernatant media was removed and 150 μl of DMSO was added to each well. After 5 minutes, the absorbance was measured at 490 nm in a microplate reader (BioTek, Winooski, VT).

### LDH assay

5-HTR1E HEK293 cells were seeded in 96 well plate (1×10^5^/well). On the next day, these cells were pretreated with 1μM 5-HT, BRL54443 overnight and then challenged with 300 μM H_2_O_2_ for 6h. Cytotoxicity was measured by the amount of LDH released using a CytoTox 96 Non-radioactive cytotoxicity assay kit according to manufacturer’s instruction (Promega, Madison, WI).

### RNA-seq analysis

5-HTR1E expressing and control HEK293 cells were seeded in a 6 well plate. On the next morning, cells were serum-starved for 3h. and then treated with 1μM 5-HT for 1h. RNA was extracted from these cells using the RNAeasy mini kit (Qiagen). Poly(A) sequencing libraries were prepared with the Illumina TruSeq stranded mRNA protocol, checking RNA quality with Agilent Technologies 2100 Bioanalyzer. Paired-end, 140-bp reads were sequenced on an Illumina NovaSeq 6000.

Paired-end fastq reads underwent quality control checks using FastQC (16), RseQC (17) (18), and MultiQC (19) both before and after trimming reads with cutadapt v3.4 (20) with arguments -q 20 and –minimum-length 25 to perform light quality trimming and retain reads with length > 25 bp following adapter trimming. After confirming all sample libraries passed quality control checks, reads were aligned to the GRCh38 human reference genome using the STAR aligner v2.7.8a (21) with arguments –outFilterType BySJout, --outFilterMultimapNmax 20, --alignSJoverhangMin 8, --alignSJDBoverhangMin 1, --outFilterMismatchNmax 999, --outFilterMismatchNoverReadLmax 0.04, --alignIntronMin 20, --alignIntronMax 1000000, --alignMatesGapMax 1000000 to match ENCODE standard options for long RNA sequencing. Reads were then counted in genes according to the GENCODE release 28 annotation using the featureCounts tool of the subread package v2.0.1 (22) in stranded mode (-s2 argument). Differential expression analysis was conducted on gene counts using DESeq2 v1.34.0 (22).

Contrasts were constructed that evaluated differences at the group level, where each group is a combination of the genotype and treatment applied for a given sample (ex: HTR1E-stable_serotonin represents HTR1E-overexpressing cells treated with serotonin). Pairwise comparisons and interaction contrasts were conducted using the model ~group, specifying contrasts using numeric vectors, and using “ashr” log2 fold change shrinkage. Genes were identified as differentially expressed if they had had an adjusted p-value < 0.1. Functional enrichment was performed with the clusterProfiler package v4.2.0. RNA-seq figures were generated using the ggplot2 v3.3.5 package in R v4.1.1.

## Results

### Gαi linked 5-HTR1E-cAMP pathway is PKA independent

**Fig. 1.**
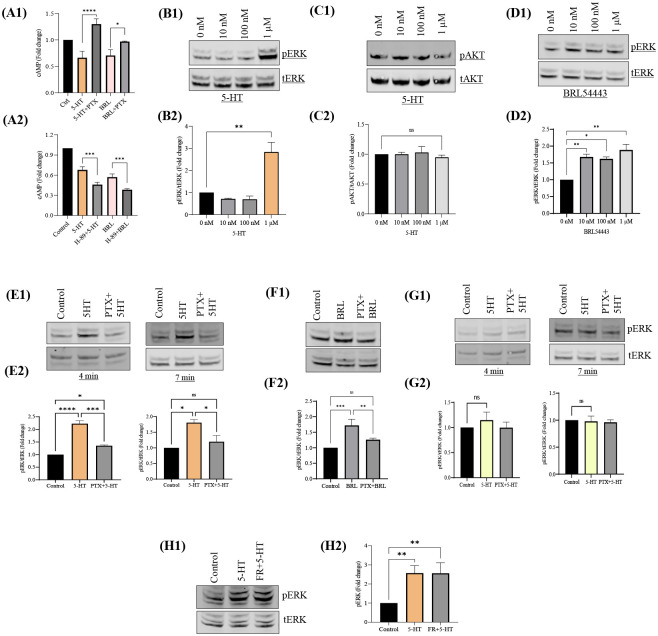
5-HTR1E-cAMP pathway is Gαi dependent and PKA independent. 5-HTR1E stable cells were incubated with 200 ng PTX **(A1)** for 24 h. or **(A2)** 10 μM H-89 (PKA inhibitor) for 30 min. After induction with 10 μM forskolin, cells were treated with 1 μM 5-HT or BRL54443(5-HTR1E agonist) for 20 min. Fold change in cAMP levels were measured by cAMP-Glo^™^ Assay kit. One-way ANOVA analysis followed by Tukey’s *post hoc* multiple comparison test, values are mean ± SD, N=3. **Serotonin activates ERK signaling *via* 5-HTR1E.** HEK293 cells stably transfected with 5-HTR1E were treated with 0 nM to 1 μM 5-HT or BRL54443 between 4 to 7 min and pERK and pAKT were analyzed by western blotting. Bar graphs showing the Image *j* quantification of blots as fold change in pERK1/2 (**B1–2), (D1–2)** and pAKT **(C1–2)** after normalization with tERK1/2 and tAKT as loading control. One-way ANOVA analysis followed by Tukey’s *post hoc* multiple comparison test. p values are mean ± SD, N=2. **Serotonin**-**5-HTR1E mediated ERK phosphorylation is Gαi dependent.** 5-HTR1E stable cells were treated with 200 ng PTX (Gi inhibitor) for 24 h. followed by treatment with 1μm 5-HT or BRL54443 for 4 to 7 min. Western blot and bar graphs showing the fold change in 5-HT **(E1–2)** and BRL54443 **(F1–2)** induced pERK1/2. **(G1–2)** Control HEK293 cells, p=ns. **(H1–2)** ERK analysis in 5-HTR1E stable cells pretreated with 1μm FR900359 (Gq inhibitor) for 30 min. followed by 1μm 5-HT induction. Values are mean ± SD, N=3. *p<0.05, **p <0.005, ***p<0.0001, ****p<0.0001.

Gαi-linked GPCRs are known to inhibit cAMP levels *via* protein kinase A (23). Since 5-HTR1E is also a Gαi dependent receptor (24) we investigated the role of PKA in 5-HTR1E mediated cAMP reduction. 5-HTR1E stable cells were pretreated with Gαi inhibitor PTX or PKA inhibitor H-89 which was followed by 1μM 5-HT or BRL54443 treatment, in the presence of forskolin. Results show that both 5-HT and BRL54443 treatment reduced the cAMP levels significantly and this decrease in cAMP was reversed by pretreatment of PTX (p<0.05) (Fig. 1, A1). In H-89 pretreated cells cAMP concentration could not be restored to normal levels and an additional decrease (~20%) was observed in cAMP level as compared to 5-HT (33%) and BRL54443 (43%) treatment alone (p<0.001) (Fig. 1, A2). These results indicate that serotonin-5-HTR1E mediated cAMP pathway is Gαi dependent, but unlike the other classical Gi-protein coupled serotonin receptors (25, 26), 5-HTR1E receptor is not protein kinase A dependent.

### Serotonin-5-HTR1E activates ERK pathway

To examine the effect of serotonin on 5-HTR1E mediated ERK and AKT signaling pathways, we treated 5-HTR1E stable HEK293 cells with 0 nM to 1μM 5-HT between 4 to 7 min. Fig. 1B shows that 1μM serotonin increased ERK phosphorylation 2.8-fold (p<0.005) (Fig. 1, B1–B2), but no increase was observed in AKT phosphorylation (Fig. 1, C1–C2). To determine if 5-HTR1E agonist BRL54443 could also activate the ERK signaling, we treated 5-HTR1E stable cells with 0 nM to 1μM BRL54443 and observed that at a lower dose of 10 nM, BRL54443 increased ERK phosphorylation significantly (1.6-fold) and highest increase (~1.9-fold) was achieved at 1μM dose (Fig. 1, D1–D2) which is similar to the 5-HT induced effect on pERK. This dose dependent effect of serotonin was also checked in control HEK293 cells but no significant increase in ERK1/2 phosphorylation was observed (Suppl. fig. S1, A1–A2). Next, we investigated the dose dependent effect of serotonin on SH-SY5Y cells with endogenous 5-HTR1E expression, but the rate and magnitude of 5-HT-induced ERK1/2 activation was considerably less (Suppl. fig. S1, panel B1–B2) in these cells compared to 5-HTR1E stable HEK293 cells. Taken together these results indicate that 1 μM serotonin or agonist (BRL54443) is the optimum dose to activate ERK pathway in 5-HTR1E overexpressing HEK293 cells while AKT pathway remains unaffected by serotonin-5-HTR1E induction in these cells.

### Serotonin induced 5-HTR1E/ERK pathway is Gαi dependent

To further explore the involvement of various Gα proteins in serotonin-5-HTR1E induced ERK phosphorylation, we examined the effect of Gαi inhibitor PTX and Gαq inhibitor FR900359 (27) on 5-HTR1E expressing and control HEK293 cells between 4 to 7 min. 1μM 5-HT or BRL54443 induced ERK phosphorylation was completely (above 80%) reversed in cells pretreated with PTX for 24 h. (Fig. 1, E1–F2) (p<0.05) as compared to the cells not pretreated with PTX (Fig. 1, G1–G2). Treatment with Gq inhibitor showed no effect on 5-HT mediated ERK phosphorylation (Fig. 1, H1–H2). These results show that serotonin-5HTR1E mediated ERK activation is Gαi dependent while Gαq is not involved in 5-HTR1E mediated ERK phosphorylation.

### Serotonin activated ERK pathway does not involve Gβϒ subunits

**Fig. 2.**
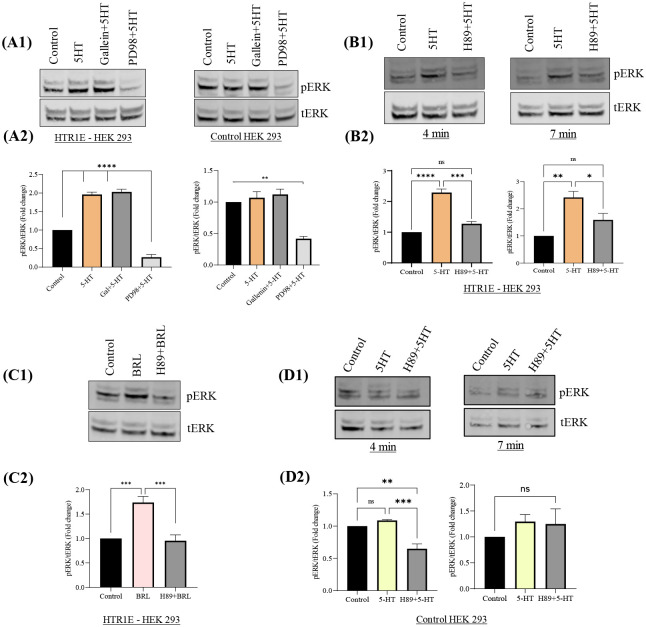
5-HTR1E mediated ERK phosphorylation is independent of Gβϒ but involves PKA activation. ERK analysis in 5-HTR1E stable cells pretreated with 10 μM Gallein (G_βϒ_ inhibitor), or 5 μM PD98059 (MEK inhibitor) **(A1–2)** or 10 μM H-89 (PKA inhibitor) **(B1–2)** for 30–45 min. followed by 1 μM 5-HT. Bar graph showing the fold change in pERK1/2. **PKA inhibitor can reverse 5-HTR1E agonist mediated pERK.** 5-HTR1E stable cells preincubated with H-89 were induced with 1 μM BRL54443. Panel **(C1–2)** showing the effect PKA inhibitor on BRL54443 activated pERK. **(D1–2)** Effect of H-89 (PKA inhibitor) in control HEK293 cells. One-way ANOVA analysis followed by Tukey’s *post hoc* multiple comparison test. values are mean ± SD, N=3. *p<0.05, **p <0.005, ***p<0.0001, ****p<0.0001.

In Gαi coupled GPCRs, Gβγ subunits have an important role in the downstream ERK signaling cascade (28–30). To determine the contributions of Gβϒ on Gαi-dependent MAP kinase activation, 5-HTR1E expressing and control HEK293 cells were pretreated with Gβϒ inhibitor Gallein (10 μM) for 30 min or PD980059, MEK inhibitor (5 μM, 45 min) prior to stimulation with 1μM 5-HT. As shown in figure 2A1–A2, the Gβϒ inhibitor failed to inhibit 5-HT-stimulated ERK1/2 phosphorylation while MEK inhibitor (positive control) totally blocked this 5-HTR1E mediated effect. Thus, our data suggest that Gβγ subunits are not required for the 5-HT-mediated ERK signaling in 5-HTR1E expressing cells.

### Serotonin-5-HTR1E stimulated ERK activity is mediated by PKA and PI3-K

**Fig. 3.**
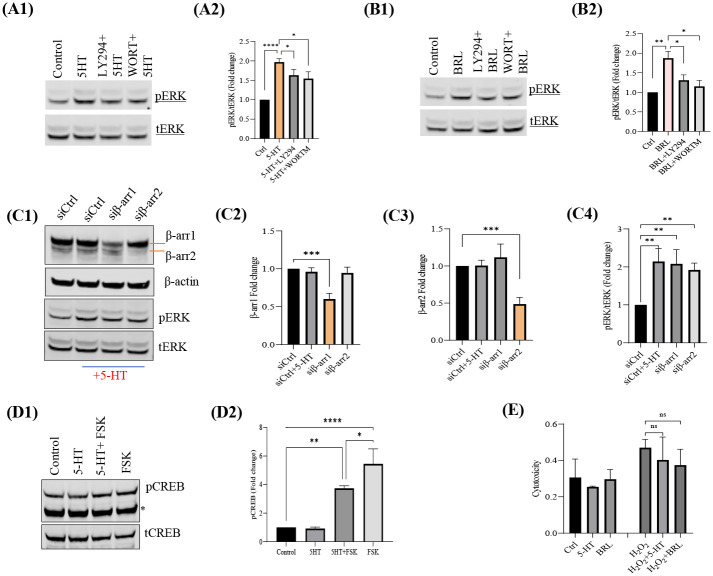
PI3K mediates serotonin-5-HTR1E induced pERK. 5-HTR1E expressing cells were incubated with 30 μM LY294002 or 0.5 μM wortmannin (PI3K/Akt inhibitor) for 30 min, followed by treatment with 1 μM 5-HT or BRL54443, and effect on pERK was assessed by western blot. Bar graph showing the effect of PI3-K inhibitors on 5-HT **(A1–2)** and BRL54443 **(B1–2)** mediated pERK. **Serotonin-5-HTR1E induced ERK activation is β-arrestin independent.** 5-HTR1E expressing HEK293 cells were transfected with 50 μM control or β-arrestin 1 and 2 siRNA. After 48 h. cells were treated with 1 μM 5-HT for 7 min. and changes in β-arrestin 1 and 2 and pERK expression were analyzed **(C1)**. Bar graphs showing the fold change expression of **(C2)** β-arrestin 1 **(C3)** β-arrestin 2 and **(C4)** pERK1/2. **5-HTR1E inhibit FSK stimulated CREB phosphorylation**. 5-HTR1E expressing cells were incubated with 10 μM FSK followed by a treatment with 1μm 5-HT for 30 min. pCREB levels were analyzed by western blotting. **(D1–2)** Bar graphs showing the fold change in pCREB after normalization with tCREB as an internal control. *ATF band which also cross-reacts with this antibody. **Lactate dehydrogenase (LDH) cytotoxicity assay. (E)** 5-HTR1E expressing HEK 293 cells were treated with 1 μM 5-HT, BRL54443 followed by 300 μM H_2_O_2_ for 6h. LDH analysis were performed using LDH cytotoxicity assay kit. One-way ANOVA analysis followed by Tukey’s *post hoc* multiple comparison test. values are mean ± SD, N=3. *p<0.05, **p <0.005, ***p<0.0001, ****p<0.0001.

Some of the GPCRs are known to exert their physiological actions through activation of Gi/PKA/PI3-K-dependent ERK signaling (29, 30). To explore the detailed mechanism behind the serotonin induced 5-HTR1E- mediated ERK pathway, we used various inhibitors against PKA and PI3-K. 5-HTR1E stable HEK293 cells were pretreated with the PKA inhibitor H89 and PI3K inhibitors, LY294002 and wortmannin, prior to stimulation with 5-HT. Results show that H-89 blocked the 5-HT (Fig. 2, B1–2) or BRL54443 (Fig. 2, C1–2) stimulated ERK phosphorylation (approx. 80%, p<0.001) as compared to control HEK293 cells (Fig. 2, D1–2). PI3-K inhibitors showed a limited (~20%, but significant, p<0.05) inhibitory effect with 5-HT (Fig. 3, A1–2) but when this suppressive effect of PI3-K inhibitors was tested on BRL54443 induced pERK, the inhibition was more potent and between 30–40% (p<0.01) decrease was noted with LY294002 or Wortmannin pretreatment (Fig. 3, B1–2). These experiments were also performed in control HEK293 cells (Suppl. Fig. S1, C1–C2)

### Serotonin-5-HTR1E activated ERK pathway is β-arrestin independent

For several GPCRs, β-arrestin 1 and 2 proteins are required for the activation of ERK pathway (31–33) and previously we also showed that NF-α1/CPE activated 5-HTR1E can increase ERK phosphorylation *via* β-arrestin recruitment (4). Here we explored if serotonin-5-HTR1E mediated ERK phosphorylation is also β-arrestin dependent. β-arrestin 1 (Fig. 3, C1, C2) and β-arrestin 2 (Fig. 3, C1, C3) were knocked down in 5-HTR1E stable HEK293 cells using siRNA (40–50% reduction, p<0.05) and these cells were then treated with 1μM 5-HT for ERK activation. Serotonin induced ERK 1/2 phosphorylation in 5-HTR1E expressing cells was not affected by β-arrestin 1 and 2 knock down (Fig.3, C1, C4). These results indicate that β-arrestin 1 and 2 proteins do not play any role in 5-HTR1E mediated ERK phosphorylation.

### 5-HTR1E inhibit forskolin stimulated CREB phosphorylation

CREB is a downstream effector of both cAMP and ERK signaling pathways (34, 35) and its phosphorylation is related to various physiological processes via serotonin receptors (36). To further investigate the effect of serotonin on 5-HTR1E mediated CREB phosphorylation, we treated 5-HTR1E stable or control HEK293 cells with 1μM 5-HT in the presence and absence of forskolin. After 20 min. of treatment, a significant decrease (31%, p<0.05) was observed in forskolin stimulated CREB phosphorylation (Fig.3, D1–2). These data show that serotonin activated 5-HTR1E is a negative regulator of CREB phosphorylation.

### Serotonin-5-HTR1E does not protect against H_2_O_2_-induced cytotoxicity

Previously, we have shown that pretreatment with NF-α1/CPE can protect against H_2_O_2_-induced cytotoxicity in 5-HTR1E expressing cells (4). Here we sought to determine if such an effect can also occur with serotonin pretreatment. 5-HTR1E stable cells were treated with 1μM 5-HT or BRL54443 for 24 h followed by 300 μM H₂O₂ treatment for 6 h. We observed that 5-HT or BRL54443 treated cells did not have decreased cytotoxicity when challenged with H_2_O_2_ (Fig.3E). Thus, the serotonin-5-HTR1E interaction does not play a role in protection of cells against H_2_O_2_-induced oxidative stress.

### 5-HTR1E regulates the expression of pERK, pAKT and cell cycle genes in SHSY-5Y cells

**Fig: 4.**
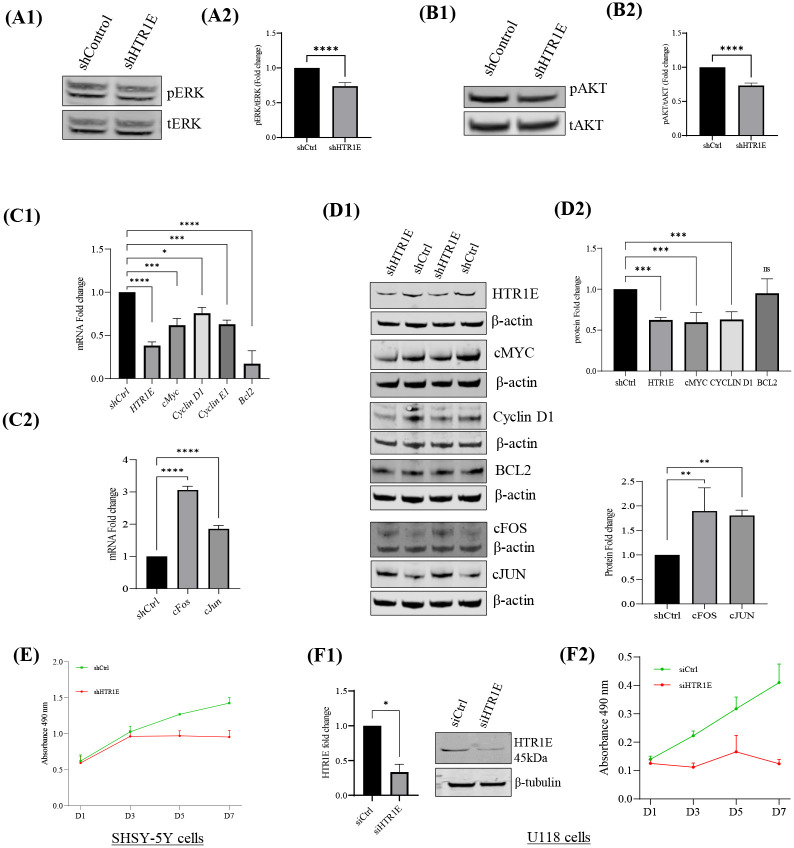
Role of 5-HTR1E in cell survival. SH-SY5Y cells were transduced with 40 moi AdHTR1E or AdControl-shRNA for 72 h. pERK and pAKT were analyzed by western blot, bar graphs showing the fold change in protein expression levels. Statistical analysis was performed using student’s t-test for **(A1–2)** pERK1/2 **(B1–2)** AKT, *p<0.0001 in shControl vs shHTR1E. Data represent mean ± SD from 3 independent experiments. **(C1–2)** show the fold change in mRNA levels of *5-HTR1E, cMyc, Cyclin D1, Cyclin E, Bcl2, cFos, cJun* gene, while representative western blot and bar graph in **(D1–2)** show the reduced levels of these proteins in SH-SY5Y cells treated with AdHTR1E-shRNA relative to cells treated with AdControl-shRNA. mRNA and protein data were normalized to internal control 18s or β-actin and are presented as mean ± SD, N=3, **Effect of 5-HTR1E knock down on SH-SY5Y and U118 cell survival.** SH-SY5Y cells **(E)** were transduced with 40 moi shHTR1E or shControl virus while U118 cells **(F1–2)** were transfected with 50 nM siHTR1E or siControl. Effect of 5-HTR1E knock down on cell survival was checked but MTT assay over the period of 7 days. *p<0.01, **p <0.001, ***p<0.0001, ****p<0.0001.

To determine the effect of 5-HTR1E KO on ERK and AKT phosphorylation in SHSY-5Y cells which have substantial endogenous 5-HTR1E, we used Ad-5HTR1E shRNA to knockdown 5-HTR1E. A very little but significant (25%, p<0.001) decrease was observed in both pERK (Fig. 4, A1–A2) and pAkt (Fig. 4, B1–B2) levels in 5-HTR1E KO cells as compared to control. We further investigated the effect of 5-HTR1E KO on early response genes *cfos, cJun*, cell cycle related genes *cMyc, Cyclin D1, Cyclin E* and pro survival *BCL2*. Fig. 4C shows that there was a marked increase in mRNA expression of both *cFos* (3-fold) and *cJun* (2-fold) after 5-HTR1E KO while *cMyc, Cyclin D1, Cyclin E* and *BCL2* genes were reduced significantly (p<0.5 to 0.001) (Fig. 4, C1–C2). We also examined the effect of 5-HTR1E KO on the protein levels of these genes and observed that the protein expression was consistent with mRNA data except for BCL2 protein which did not show any reduction (80% decrease in mRNA) after 5-HTR1E knockdown (Fig. 4, D1–D2).

### 5-HTR1E mediates cell survival

Since 5-HTR1E knock-down caused a reduction in cell cycle related genes (Fig 4, C–D), we examined the role of 5-HTR1E in cell survival. The effect of 5-HTR1E knock-down on survival of SHSY-5Y neuroblastoma and U118 glioblastoma cells which express large amounts of 5-HTR1E was investigated by MTT assay. SHSY-5Y treated with Ad-5-HTR1E shRNA reduced the expression of 5-HTR1E significantly (P<0.01) and MTT analysis showed more than 2-fold decrease (p<0.001) in survival in 5-HTR1E knockout cells from day 5 to day 7 (Fig.4E). In U118 cells treated with siRNA, 5-HTR1E expression was reduced ~65% (Fig. 4F1). MTT analysis (Fig. 4F2) over a 7-day period showed a significant decrease (~3-fold inhibition on day 7) in U118 cell survival after 5-HTR1E knock-down, compared to control cells. These data indicate that 5-HTR1E plays a key role in promoting survival of SHSY-5Y and U118 cells.

### Profiling of 5-HTR1E-regulated gene expression by RNA-seq analysis

**Fig: 5.**
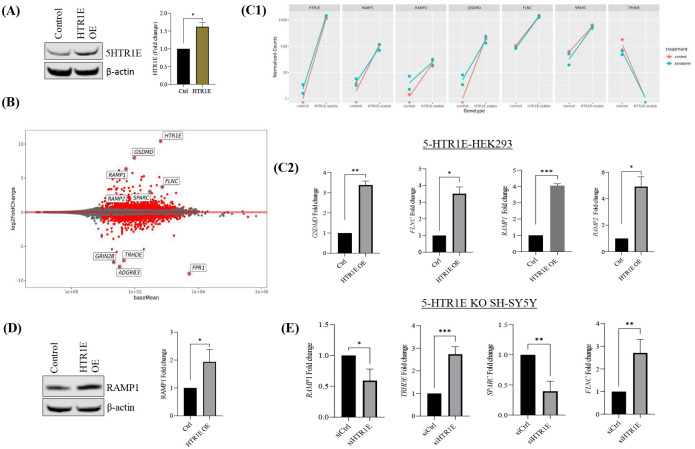
RNA-seq analysis in 5-HTR1E overexpressing HEK293 cells. **(A)** 5-HTR1E overexpression in HEK293 cells. **(B)** MA plot of RNA-seq analysis showing up and downregulated genes in 5-HTR1E overexpressing in HEK293 cells. Effect of 5-HTR1E overexpression on target genes in **(C1)** RNA-seq and **(C2)** qRT-PCR **(D)** RAMP1 protein expression in 5-HTR1E overexpressing HEK293 cells. **(E)** Effect of 5-HTR1E knock down on target gene expression in SH-SY5Y cells. Statistical analysis was performed using student’s t-test, mRNA and protein data were normalized to internal control 18s and β-actin. Results are presented as mean ± SD, N=2, *p<0.01, **p <0.001, ***p<0.0001, ****p<0.0001.

To gain further insight into how 5-HTR1E overexpression (Fig. 5A) with or without serotonin can induce changes in gene expression profile of HEK293 cells, RNA-seq analysis was performed. 5-HTR1E overexpressing and control HEK293 cells were treated with or without 1μM 5-HT and mRNA was subjected to RNA-seq analysis. 594 genes were found to be up-regulated in 5-HTR1E overexpressing samples at a statistically significant level (FDR<0.1) while 551 genes were down-regulated (Fig.5B). Modeling the interaction term between overexpression status and 5-HT treatment revealed a total of 229 genes that had a 5-HTR1E-specific response to 5-HT. For example, genes that were unresponsive to 5-HT only when 5-HTR1E was overexpressed tended to be related to innate immune response, chromatin assembly, and clathrin binding. Top genes affected by serotonin interaction in 5-HTR1E overexpressing cells included Acid-sensing ion channel 3 (*ASIC3*), RNA Component of Signal Recognition Particle 7SL 2 and 3 (*RN7SL2 and RN7SL3*) lecithin-cholesterol acyltransferase (*LCAT*), Reticulocalbin-3 (*RCN3*), Anti-Mullerian Hormone (*AMH*), Kinesin Family Member C2 (*KIFC2*,) Tropomyosin 2 (*TPM2*) which are shown in supple. fig. S3–S4.

RNA-seq library preparation, by necessity, uses a single PCR cycle count for all transcripts but this may not be optimal especially for low-expression genes. Therefore, some of the differentially expressing genes were analyzed by qRT-PCR in 5-HTR1E overexpressing HEK293 and 5-HTR1E KO SHSY5Y cells. Gasdermin D (*GSDMD*), the second-most upregulated gene after 5-HTR1E itself was increased over 200x (logFC 7.96) in the HTR1E overexpressing cells compared to control. *GSDMD* is involved in immune response (37) and the functional enrichment analysis for the interaction term shows evidence of modifying the antimicrobial response (38).

Filamin C (*FLNC*) which is an actin-binding protein involved in cardiomyopathy (39) was increased by 3.7 log2FC. Secreted protein acidic and rich in cysteine (*SPARC*) which is also known as Osteonectin binds to the calcium in the bone and initiate mineralization and crystal formation (40). SPARC expression was 2.99 logFC up in 5-HTR1E overexpressing cells. Thyrotropin Releasing Hormone Degrading Enzyme (*TRHDE*) which is a pyroglutamyl- peptidase II enzyme was downregulated −7.063 logFC. 5-HTR1E mediated effect on the expression of these genes is shown in Fig. 5, C-E. Additionally, Receptor activity-modifying proteins, nuclear receptor and cyclins were further studied as described below.

### RAMP1 expression is regulated by 5-HTR1E

Receptor activity-modifying proteins (RAMPs) which were originally identified for modifying the activity of Calcitonin receptors, are now known to interact with many GPCRs (41). Our RNA-seq results showed a 6.3-fold (log2FC) (Fig. 5B and C) increase in *RAMP1* gene expression in 5-HTR1E overexpressing cells as compared to control HEK293. We examined the effect of 5-HTR1E overexpression on *RAMP1* by qPCR and western blot and found a ~ 4-fold mRNA (Fig.5, C2) and 2-fold increase in RAMP1 protein (Fig. 5, D1–2) in 5-HTR1E expressing cells, as compared to the control HEK293. We also checked the *RAMP1* mRNA expression in 5-HTR1E KO SH-SY5Y cells and observed that *RAMP1* gene expression was significantly (p<0.05) decreased after 5-HTR1E KO (Fig. 5E). In a reverse experiment, we inhibited RAMP1 expression in SH-SY5Y cells using siRNA and determined if it has any effect on 5-HTR1E. We did not observe any significant change in 5-HTR1E expression (Supple. Fig. S2, A1–2) which suggests that 5-HTR1E is upstream of RAMP1 and can regulate its expression at both gene and protein level.

### Serotonin can induce nuclear receptors, Cyclins and Cyclin Dependent kinases *via* 5-HTR1E

**Fig: 6.**
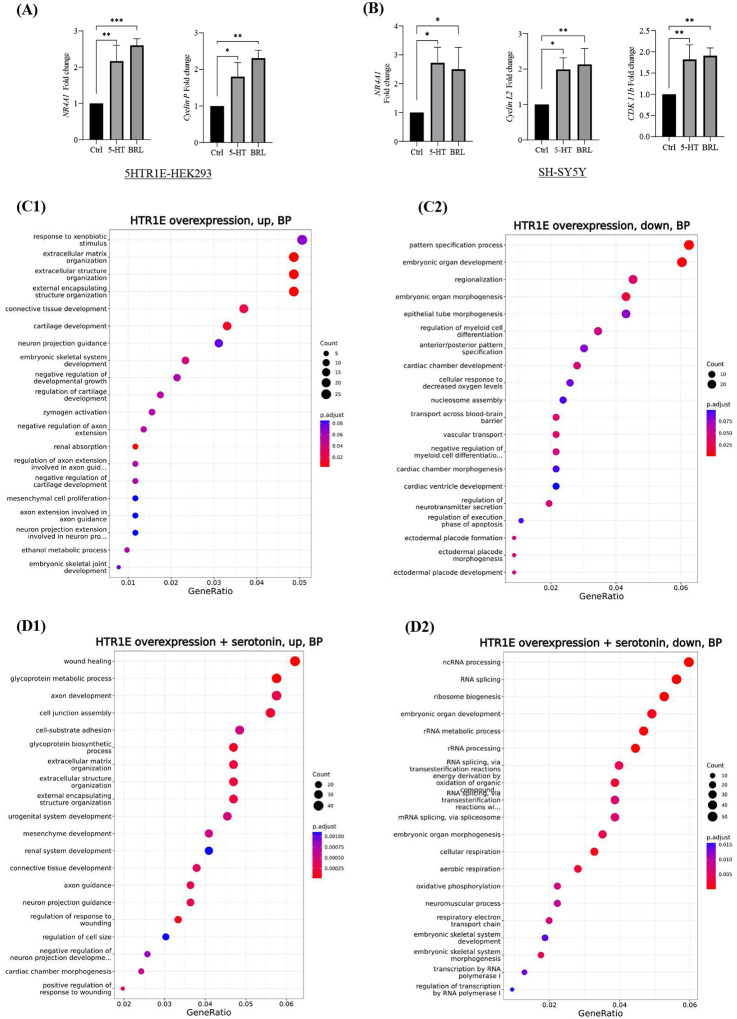
**(A)** mRNA expression of *NR4A1* and *Cyclin P* after 5-HT and BRL54443 treatment in 5-HTR1E overexpressing HEK 293 cells. **(B)** mRNA expression of *NR4A1, Cyclin L2* and *CDK11b* after 5-HT and BRL54443 treatment. Pathway analysis using ‘GO’ biological process in 5-HTR1E overexpressing HEK 293 cells **(C1-C2)** without 5-HT **(D1-D2)** in the presence of serotonin.

Based on the RNA-seq results, we also tested various genes including *Cyclin P* and *Cyclin L* by qRT-PCR. 5-HTR1E overexpressing HEK293 cells were treated with 5-HT or BRL54443 for 90 minutes and expression of *NR4A1* (or *NURR77*), *Cyclin P, Cyclin L, CDK10* and *CDK11b* were examined. In HEK293 cells *NR4A1* and *CCNP (CNTD2)* gene expression was increased almost 2-fold (Fig. 6A) in both 5-HT or BRL54443 treated groups while *Cyclin L2* and *CDK11b* were significantly increased only in BRL54443 groups (Suppl. Fig. S2, B1–B2). When SH-SY5Y cells with endogenous 5-HTR1E expression were treated with 5-HT or BRL54443, expression of *NR4A1* mRNA increased up to 3-fold while *Cyclin L2* and *CDK11b* genes were almost 2-fold higher as compared to untreated SH-SY5Y cells (Fig. 6B). These data show that 5-HTR1E can induce the expression of *NR4A1*, *CDK11b*, *Cyclin P* and *Cyclin L* genes which are involved in various physiological processes (42, 43).

### Pathway analysis

We also analyzed the pathways changed in response to 5-HTR1E overexpression and serotonin treatment using functional enrichment focusing on the Biological Process (BP) ontology. The pathways enriched in up-regulated genes were related to extracellular matrix and structure organization which also have been reported previously (9) as well as pathways related to axon regulation, connective tissue development and renal absorption (Fig. 6, C1). Pathways enriched in genes down-regulated by 5-HTR1E overexpression were related to embryonic organ morphogenesis and development, pattern specific process and transport across blood brain barrier (Fig.6, C2). Upon serotonin treatment in 5-HTR1E expressing cells, pathways related to wound healing, glycoprotein metabolism, axon and mesenchymal development were most significantly upregulated (Fig. 6, D1) while down-regulated pathways were mostly related to RNA splicing, RNA metabolism and ribosome biogenesis (Fig. 6, D2).

## Discussion

Previously our laboratory reported that 5-HTR1E, a member of the serotonin receptor family, has a robust effect in promoting neuronal cell survival during oxidative and neurotoxic stress *via* extracellular interaction with CPE/NFα1 (4). In the present study, we investigated the molecular mechanisms associated with the activation of cAMP and ERK signaling cascades by the human 5-HTR1E in response to its natural ligand, serotonin. Our data suggest that serotonin-stimulated 5-HTR1E promotes Gαi activation which in turn inhibit cAMP and stimulate ERK1/2 phosphorylation by two independent signaling mechanisms. 5-HTR1E has a classic cAMP inhibition function like other 5-HT1 family members but the mechanism of this serotonin-5-HTR1E mediated cAMP inhibition had not been studied in detail. Here we report that the effect of serotonin-5-HTR1E on cAMP is through Gαi (Fig. 1, A1), but it does not involve PKA (Fig. 1, A2). Moreover, 5-HTR1E mediated cAMP inhibition is exclusive to its serotonin binding pocket as only 5-HT or BRL54443 showed an inhibitory effect on cAMP but not CPE/NFα1 which interacts with extracellular domains of 5-HTR1E *via* H-bonds and salt bridges (4). To further explore the effect of 5-HTR1E mediated cAMP inhibition we determined the effect of serotonin on CREB phosphorylation which is a downstream effector of the cAMP pathway (44), and observed that serotonin can reduce the level of forskolin stimulated pCREB significantly (p<0.05) (Fig.3, D1–2). These results show that Gαi linked 5-HTR1E can inhibit cAMP and pCREB *via* serotonin activation.

To analyze the other downstream signaling pathways of 5-HTR1E, we examined the dose dependent effect of 5-HT on 5-HTR1E expressing HEK293 cells at different concentrations and found that 1 μM 5-HT or BRL54443 could induce ERK phosphorylation significantly (Fig. 1). Using specific inhibitors, we found that this serotonin induced ERK activation is Gαi, PKA and PI3K dependent and it does not involve Gβϒ and Gq proteins (Fig. 1 and 2). Previously, we have also shown that the physiological effects of 5-HTR1E stimulation in response to CPE/NFα1 resulted from β-arrestin-dependent ERK activation (4). To evaluate any involvement of β-arrestin in serotonin-induced ERK pathway we used siRNA to knockdown β-arrestin 1/2 protein. We observed that the reduction in β-arrestin expression levels had no impact on 5-HT induced ERK pathway (Fig. 3, C1–2). Also, the late ERK activity (7–10 min) of CPE/NFα1–5-HTR1E was found to be independent of Gαi (4), while it was much faster in the case of serotonin-5-HTR1E (4–7 min). Moreover, using 5-HTR1E-HEK293 and human neurons, we showed that NF-α1/CPE can protect against H_2_O_2_- induced cytotoxicity (4), but this protective effect could not be achieved with serotonin-5-HTR1E interaction (Fig. 3E), and is therefore an exclusive function of NF-α1/CPE-mediated activation of 5-HTR1E. These results show that both serotonin and CPE/NFα1 follow two distinct mechanisms for 5-HTR1E mediated ERK activation. In addition to serotonin induced function in 5-HTR1E overexpressing HEK293, we also found some important effect of 5-HTR1E KO in SHSY-5Y cells which express a high amount of 5-HTR1E. Fig. 4 shows that in 5-HTR1E KO SHSY-5Y cells there was a small but significant decrease in ERK and AKT phosphorylation, which was followed by further reduction in cell cycle related genes *cMyc, cyclin D1, cyclin E* and pro-survival *BCL2*. These genes are essential for cell cycle (45) and our results indicate that the expression of 5-HTR1E is important for regulation of these genes/proteins and is crucial for cell cycle. Moreover, MTT assay in 5-HTR1E KO SHSY-5Y and U118 cells showed reduced survival/proliferation of these cells in the absence of 5-HTR1E which highlights its physiological role in these processes.

Interestingly, 5-HTR1E knock-down activated ERK *via* pSRC in ovarian cancer cells, but inhibited cell proliferation (9) while in HEK293 its overexpression activates ERK in a ligand-dependent manner and is important for survival of SHSY-5Y and U118 cells. All these data and report suggest that 5HT1 receptors have unique coupling mechanism(s) in different cell and tissue types. Our study highlights the 5-HTR1E signaling mechanism specific for serotonin induction in human HEK293, SHSY-5Y and U118 cells.

To gain further insight into the genes and pathways regulated by 5-HTR1E with and without serotonin induction we performed RNA-seq analysis in 5-HTR1E expressing HEK293 cells. Various genes related to the important biological processes were changed in response to 5-HTR1E expression (Fig. 5), notably human RAMP family members which are single span transmembrane proteins and can modulate functions of several GPCRs (41). Serotonin treatment increased the expression of *CDK 11b*, a serine/threonine protein kinases and *Cyclin L2, Cyclin P* genes which control kinase activity of CDKs (43, 46). *NR4A1* which takes part in several biological processes such as cell proliferation, apoptosis (42) was also increased after serotonin treatment. Pathway analysis revealed that 5-HTR1E overexpression can affect several biological processes (Fig. 6). Change in the expression of several important genes and pathways shows that 5-HTR1E might regulate various biological functions upon induction with its ligand serotonin and further exploration of this RNA-seq data may provide detailed information about the specific physiological role of 5-HTR1E in regulation of these pathways.

## Conclusions

**Fig: 7.**
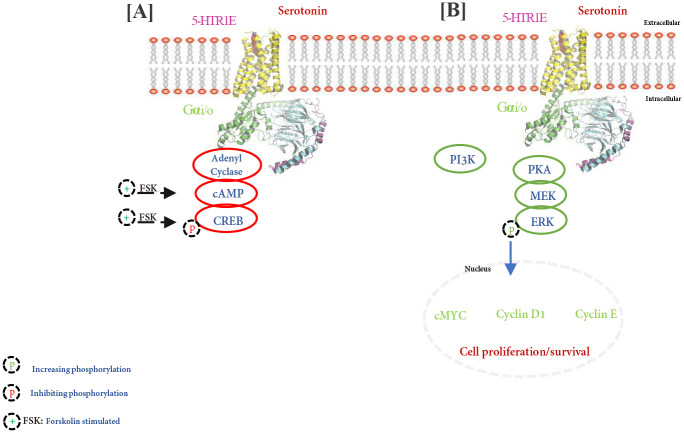
Schematic representation of human 5-HTR1E signaling. **S**erotonin stimulated 5-HTR1E receptor simultaneously activates cAMP and ERK pathways *via* Gαi. Upon serotonin treatment 5HTR1E recruits Gαi protein to its intracellular domains and **[A]** inhibit cAMP levels *via* inhibition of adenyl cyclase which further downregulate forskolin stimulated pCREB. On the other hand, serotonin-5-HTR1E **[B]** increase ERK phosphorylation *via* Gαi/PKA/PI3-K which regulate the expression of cyclin and nuclear receptor genes and plays a role in the survival of various cells.

Our results demonstrated that serotonin stimulation of 5-HTR1E can induce signals in HEK-293 cells by at least two separate pathways: one is Gαi dependent and PKA-independent cAMP pathway (Figure 7A), and another is pERK pathway which is Gαi/PKA/PI3-K-dependent and β-arrestin-independent (Figure 7B). We also found that 5-HTR1E can regulate the expression of various genes and pathways and has a very important role in cell survival.

## Supplementary Material

1

## Data Availability

The data sets generated during and/or analyzed during the current study are available from the corresponding author on reasonable request.
