# Supplementary material for: Characterization of serotonin-5-HTR1E signaling pathways and its role in cell survival
Source: Res Sq. 2023 Jan 27:rs.3.rs-2518076. Preprint. [Version 1] doi: 10.21203/rs.3.rs-2518076/v1 (PMC9928056; doi:10.21203/rs.3.rs-2518076/v1)
Supplement: 1 [file NIHPPRS2518076V1-supplement-1.pdf]

**Supple. fig. S1**

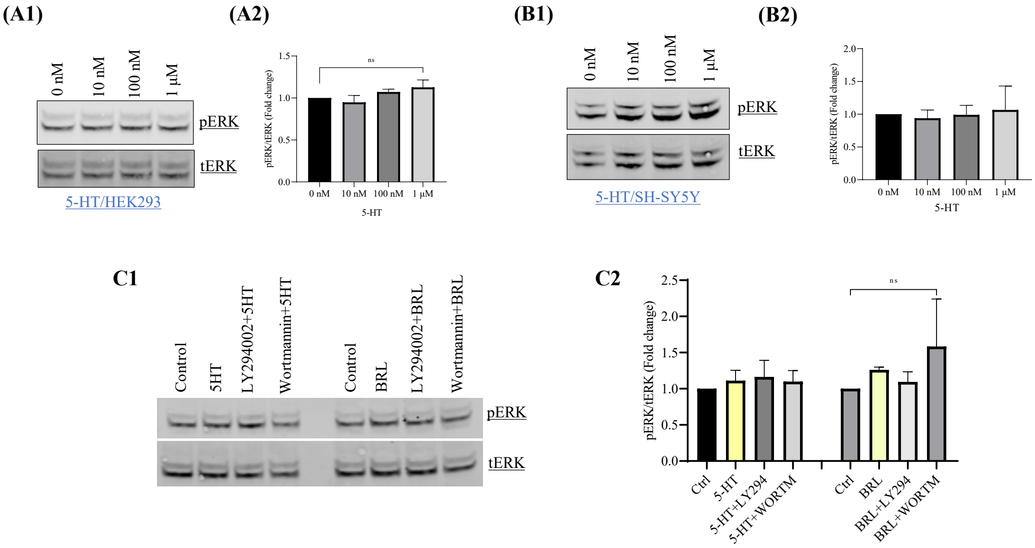


**(A1-2)** Control HEK293 cells and **(B1-2)** SHSY-5Ycells were treated with 0 nM to 1 μM 5-HT and changes in pERK 1/2 were analyzed by western blotting. Bar graphs showing the Image J quantification of blots as fold change in pERK1/2, p=ns, values are mean ± SD, N=2. **(C1-2)** Control HEK293 cells were incubated with 30 μM LY294002 or 0.5 μM wortmannin (PI3K/AKT inhibitor) for 30 min, followed by 1 μM 5-HT or BRL54443 and effect on pERK was analyzed. Bar graph showing the effect of PI3-K inhibitors on 5-HT and BRL54443 mediated pERK. p=ns, values are mean ± SD, N=3.

**Supple. fig. S2**

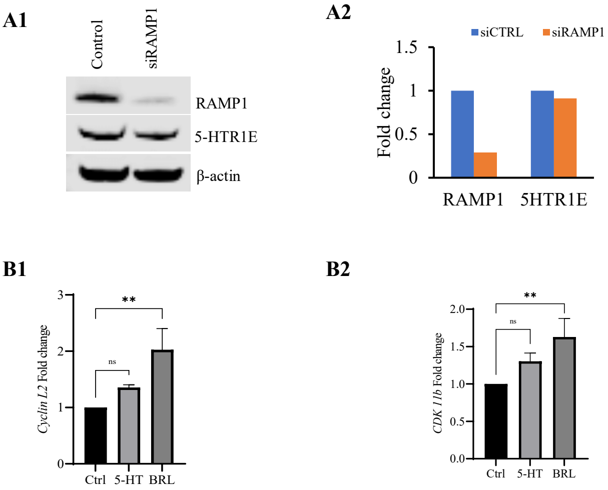


**Fig. S2. (A1-A2)** Effect of RAMP1 knock down on 5-HTR1E expression, p=ns. **(B1-B2)** Expression analysis of Cyclin L2 and CDK11b in 5-HTR1E overexpressing HEK293 cells.

**Supple. fig. S3**

Top 10 significant genes showing serotonin interaction in 5-HTR1E expressing HEK293 cells

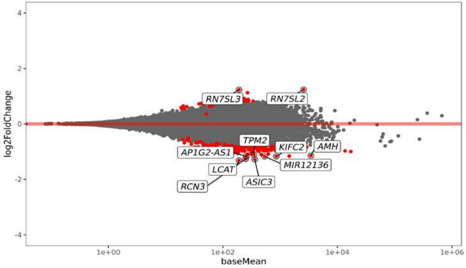


| Gene | Name/Symbol | Base Mean | Log2 fc | P value | P adj |
|---|---|---|---|---|---|
| ENSG00000213199 | Acid-sensing ion channel 3 (ASIC3) | 358.7 | -1.276 | 0.000001474 | 0.005012 |
| ENSG00000278771 | RNA Component Of Signal Recognition Particle 7SL3 (RN7SL3) | 191.0 | 1.233 | 9.614e-7 | 0.005012 |
| ENSG00000213398 | lecithin-cholesterol acyltransferase (LCAT) | 190.6 | -1.313 | 8.673e-7 | 0.005012 |
| ENSG00000142552 | Reticulocalbin-3 (RCN3) | 249.5 | -1.265 | 0.000001122 | 0.005012 |
| ENSG00000210151 | MIR12136 | 536.2 | -1.170 | 0.000002820 | 0.007670 |
| ENSG00000274012 | RNA Component Of Signal Recognition Particle 7SL2 (RN7SL2) | 2,565 | 1.240 | 0.000003627 | 0.008220 |
| ENSG00000104899 | Anti-Mullerian Hormone (AMH) | 3,436 | -1.143 | 0.000006156 | 0.01196 |
| ENSG00000167702 | Kinesin Family Member C2 (KIFC2) | 856.8 | -1.168 | 0.000007713 | 0.01311 |
| ENSG00000198467 | Tropomyosin 2 (TPM2) | 348.1 | -1.105 | 0.000008752 | 0.01322 |
| ENSG00000258727 | AP1G2 antisense RNA 1 (AP1G2-AS1) | 255.7 | -1.166 | 0.00001203 | 0.01487 |

**Supple. fig. S4**

Top 10 genes w/interaction, when serotonin is present

Exhibits more positive effect

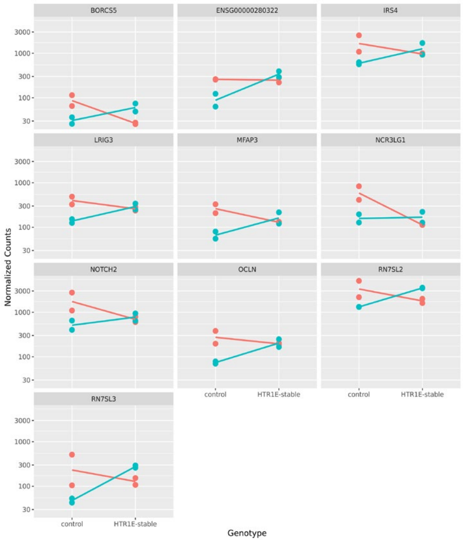


Exhibits more negative effect

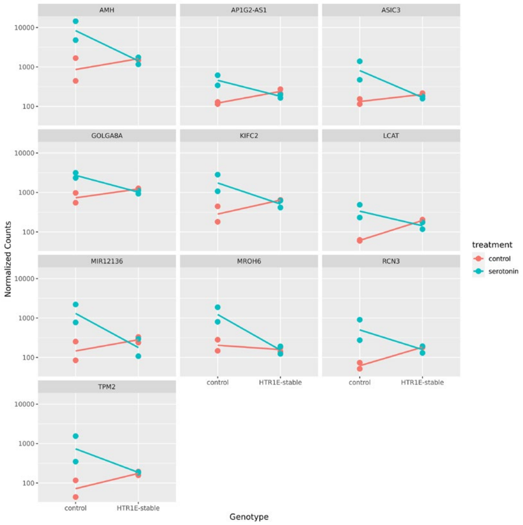

**Supplementary table 1:** List of qPCR primers

| S. No. | Gene name | Primer sequence |
|---|---|---|
| 1 | *5-HTR1E* | GGCTGCCATTTTTCATCAAAGAGTT<br>ATGAGGCTCTGGAAAAAGTCGT |
| 2 | *cFOS* | TTACTACCACTCACCCGCAGACTC<br>TGGAGTGTATCAGTCAGCTCCCTC |
| 3 | *cJUN* | CCA AAG GAT AGT GCG ATG TTT<br>CTG TCC CTC TCC ACT GCA AC |
| 4 | *cMYC* | CCTGGTGCTCCATGAGGAGAC<br>CAGACTCTGACCTTTTGCCAGG |
| 5 | *BCL2* | ACAGGGTACGATAACCGGGA<br>TGACTTCACTTGTGGCCCAG |
| 6 | *CYCLIN D1* | ACCTGGATGCTGGAGGTCTG<br>GAACTTCACATCTGTGGCACA |
| 7 | *CYCLIN E* | GGTGAGGAGCCCACTGGGGA<br>ACTTGCTGCTTCGGCCTTGT |
| 9 | *GAPDH* | GAGCCACATCGCTCAGACAC<br>CATGTAGTTGAGGTCAATGAAGG |
| 10 | *RAMP 1* | CACCCAGTTCCAGGTAGACA<br>CACTGCCAGGAAGAACCTGT |
| 11 | *TRHDE* | GGACAGTTGCAAAAGGCTTC<br>TGACAGAAAACGATGGCAAA |
| 12 | *SPARC* | CCGGGACTTCGAGAAGAACT<br>CTCATCCAGGGCGATGTACT |
| 13 | *FILAMIN C* | CCAACATCACCGACAACAAG<br>CTCCAGCATCTTTGGTGACA |
| 14 | *RAMP2* | GGGGGACGGTGAAGAACTAT<br>GTTGGCAAAGTGGATCTGGT |
| 15 | *NR4A1*<br>*(NUR77)* | TCTGCTCAGGCCTGGTGCTAC<br>GGCACCAAGTCCTCCAGCTTG |
| 16 | *NR4A2* | AGT CTG ATC AGT GCC CTC GT<br>TAT GCT GGG TGT CAT CTC CA |
| 17 | *GSDMD* | AGCCCTACTGCCTGGTGGTTAG<br>CCTGCGATCTTTGCCTGTCCTG |
| 18 | *CYCLIN L2* | GACCTCGAGTAGCTCTGCTC<br>CAGGTGCTGGATGGTACCTC |
| 19 | *CDK11b* | ACGTAGGCATCGTAGCCATT<br>CTTCCTTTCTAACTGCTCCA |
| 20 | *CYCLIN P*<br>*(CNTD2)* | CTGGTGGTAGACTGGCTGGT<br>AGCACGCACTCTTCCATTTT |

**Supplementary table 2:** List of antibodies

| S. No. | NAME | CATALOGUE NO. | Dilution |
|---|---|---|---|
| 1 | HTR1E S31 | ab154813, abcam | 1:5000 |
| 2 | cFOS | #2250T, cell signaling | 1:2500 |
| 3 | cJun | ab40766, abcam | 1:5000 |
| 4 | cMYC | #5605, cell signaling | 1:5000 |
| 5 | Cyclin D1 | #2978, cell signaling | 1:2500 |
| 6 | BCL2 | #4223, cell signaling | 1:5000 |
| 7 | RAMP1 | ab156575, abcam | 1:5000 |
| 8 | pERK Thr202/Tyr204) | #4696, cell signaling | 1:5000 |
| 9 | tERK | #9101, cell signaling | 1:5000 |
| 10 | pCREB (Ser133) | #9198, cell signaling | 1:5000 |
| 11 | tCREB | #9104, cell signaling | 1:5000 |
| 13 | pAKT (Ser473) | #4051, cell signaling | 1:5000 |
| 13 | tAKT | #4691, cell signaling | 1:5000 |
| 14 | β-Arrestin 1/2 (D24H9) | # 4674, cell signaling | 1:2500 |
| 15 | β-tubulin | # 2128, cell signaling | 1:5000 |
| 16 | β-actin Rb | #4970, cell signaling | 1:5000 |
| 17 | β-actin mo | #3700, cell signaling | 1:5000 |
| 18 | IRDye® 800CW Rb 2° Ab | P/N: 926-32213 | 1:10000 |
| 19 | IRDye® 680RD mo 2° Ab | Selected P/N: 926-68072 | 1:10000 |
